# By the Way

**Published:** 1911-04-15

**Authors:** 


### By the Way.

### MOLIERE v. MEDICINE.

Many explanations have been given of Molière's hatred for medicine. Suffering as he did from an affection of the heart, it may be that he hated the faculty because of its powerlessness to give him relief. In any case his aversion increased after assisting at the death of Gassendi, who succumbed to the effects of thirteen successive bleedings. It is also said that domestic misfortune was the origin of his dislike of doctors. Whatever be the cause, his antipathy for the medical profession was prodigious and unjustified, for even granting that medicine at his time was often hand in glove with sorcery, and allowing that charlatans often obtained good results at the expense of legitimate practitioners, it is nevertheless true that among the ranks of the latter were to be found many who accomplished great things in the matter of medical progress. It was at this period that chemistry, having rid herself of occultism, was for the first time taught in public, that Bartholin discovered new glands and renewed our knowledge of the blood, that Malpighi prosecuted his researches into all manner of things, that Leuwenhoeck recognised the blood corpuscles, and that Charas gave himself up to dangerous experiments while searching for a remedy against the bite of vipers. All this scientific endeavour, serious and useful as it was, Molière lost sight of, and his error has brought about large damage to the popularity of medicine, for his types of charlatans have become immortal, and it has needed the passage of two hundred years before it became possible to talk to doctors on the stage in language other than that employed by M. Purgon.—*La Belgique Médicale.*

### A MILITARY SUICIDE CLUB.

The German Press has recently announced the existence of a club whose members are soldiers adept in the art of suicide. The honour of possessing such an institute belongs to the garrison of Nuremburg, though its future would seem to be somewhat precarious owing to each of its members having sworn to put an end to his life. It must not, however, be imagined that Germany has anything in the nature of a monopoly as regards these exalted associations, for it has been shown again and again that true epidemics of suicide spring up at times in various countries and among both sexes. It has been thought that atmospheric conditions may have something to do with the production of such a tendency, but it is far more probable that it is the result of a spirit of imitativeness. The women of Lyons in the last century were possessed of an impulse to kill themselves by jumping into the city wells. In 1813 a woman hanged herself at Saint-Pierre-Mouzan in le Valais, and in consequence a large number of other women followed her example, and had not the authorities intervened, the epidemic bade fair to attack every female in the district. It is curious to note that attempts at interference came solely from the authorities, husbands deeming it the wiser plan to offer no contradiction to their spouses on this occasion! Napoleon ordered a sentry-box to be burnt in which several soldiers had committed suicide. Imitative self-destruction would seem to have a peculiar fascination for the military, and the story is told that on one of the soldiers of the Invalides hanging himself from a pillar, his example was imitated by a dozen of his companions. The temptation only ceased on removal of the column. Panurge's sheep have existed for all time and in all classes of society. It is impossible to distinguish the force which drives the soldier to give himself up to death in this fashion. He kills himself without a motive for no reason, simply for the fun of the thing. The cause of this impulse to self-destruction is incomprehensible: it is not lack of money, nor yet a passionate but unrequited love. In such an event as this last one could understand even if one did not entirely sympathise with the motive of despair.—*Journal de Médecine de Bordeaux.*

### SOME LATIN RECIPES.

Cato the Elder, or the Censor, celebrated for his military, judicial, and oratorical talents, for the purity of his morals, and for his hatred of luxury, wrote for his son a manual of rural economy. In it he lays bare all the precepts of agricultural science, from the art of choosing the land and its farmer to that of the best method of cultivating asparagus. A goodly number of recipes are also to be found in it which, in spite of their age, are still of use, especially the one which deals with the making of Carthaginian porridge. "Crush in water a pound of groats, add to this three pounds of cheese and a pound of honey; cook the lot in a new saucepan, and serve." The author, who cannot be suspected of undue tenderness towards Carthage, assures us that it is excellent. The cabbage, according to Cato, is the first of vegetables, and the most digestible. Take it as an opening medicine, preserved in vinegar, and you will be able to eat and drink as much as you like. Take five leaves of it after dessert, and you will be able to resume the feast. Its uses are legion, its cures marvellous. It will become, as you wish, an emetic, a purgative, a soporific, or a remedy against gout. Whoever has eaten of it will furnish liquid enough for a bath such as will invigorate children and remove from matrons all unpleasant exhalations. Cato recommends absinthe, but not as a beverage. One should carry a bouquet of it in front of one if one wishes to avoid the excoriations which at times delay the progress of the pedestrian. Have you a dislocated member? Take a green reed, cut it in the middle, and let two men hold it on your thighs while you sing "Dories, dardaries, astataries, dissunapiter." In case of a fracture say "Huat, hanat, haut ista, pista sista domiabo damnaustra," or, better still, "Huat hanat haut ista sis tar sis ardannabon damnaustra." Those who deny the practical utility of Latin can obviously never have read Cato.—*La Belgique Médicale.*

